# DNAzyme-Amplified Electrochemical Biosensor Coupled with pH Meter for Ca^2+^ Determination at Variable pH Environments

**DOI:** 10.3390/nano12010004

**Published:** 2021-12-21

**Authors:** Hui Wang, Fan Zhang, Yue Wang, Fangquan Shi, Qingyao Luo, Shanshan Zheng, Junhong Chen, Dingzhen Dai, Liang Yang, Xiangfang Tang, Benhai Xiong

**Affiliations:** 1 State Key Laboratory of Animal Nutrition, Institute of Animal Science, Chinese Academy of Agricultural Sciences, Beijing 100193, China; wanghui_lunwen@163.com (H.W.); zhangfan19@139.com (F.Z.); wangyue9313@163.com (Y.W.); luoqingyao@caas.cn (Q.L.); zhengshanshan@caas.cn (S.Z.); 2 College of Animal Science and Technology, China Agricultural University, Beijing 100193, China; 3 College of Animal Science and Technology, Northwest A&F University, Yangling 712100, China; 4 Animal Husbandry and Veterinary Station of Xihe County, Longnan 742100, China; sfquan@163.com; 5 Department of Animal Science and Technology, Jinling Institute of Technology, Nanjing 211169, China; chenjunhong@jit.edu.cn (J.C.); dzdai@163.com (D.D.)

**Keywords:** electrochemical sensor, single-walled carbon nanotube, DNAzyme, dairy cow, hypocalcemia

## Abstract

For more than 50% of multiparous cows, it is difficult to adapt to the sudden increase in calcium demand for milk production, which is highly likely to cause hypocalcemia. An electrochemical biosensor is a portable and efficient method to sense Ca^2+^ concentrations, but biomaterial is easily affected by the pH of the analyte solution. Here, an electrochemical biosensor was fabricated using a glassy carbon electrode (GCE) and single-walled carbon nanotube (SWNT), which amplified the impedance signal by changing the structure and length of the DNAzyme. Aiming at the interference of the pH, the electrochemical biosensor (GCE/SWNT/DNAzyme) was coupled with a pH meter to form an electrochemical device. It was used to collect data at different Ca^2+^ concentrations and pH values, and then was processed using different mathematical models, of which GPR showed higher detecting accuracy. After optimizing the detecting parameters, the electrochemical device could determine the Ca^2+^ concentration ranging from 5 μM to 25 mM, with a detection limit of 4.2 μM at pH values ranging from 4.0 to 7.5. Finally, the electrochemical device was used to determine the Ca^2+^ concentrations in different blood and milk samples, which can overcome the influence of the pH.

## 1. Introduction

Calcium is an essential macronutrient in living organisms that plays an irreplaceable role in physiological and biochemical functions [1,2]. Calcium ions (Ca^2+^) can maintain the biological potential on both sides of the cell membrane and normal nerve conduction function [3,4]. For dairy cows, a tremendous amount of calcium is required every day for daily milk production. To maintain the balance of calcium in the blood, dairy cows must be supplemented with a certain amount of calcium from food or absorb it from the intestines and the kidneys [5]. Inappropriate Ca concentrations affect the function and motility of the rumen, abomasum, intestines, and uterus, with severe consequences on energy metabolism [6]. At the onset of lactation, more than 50% of multiparous cows have difficulty adapting to the sudden increase in calcium demand by the mammary gland for milk production [7,8], which might cause subclinical hypocalcemia without any symptoms [9].

In general, the blood calcium concentration of healthy dairy cows is 2.1 ~ 2.5 mM, of which the ionic calcium occupies about 50%. When the blood Ca^2+^ is in the range of 0.7 ~ 1.05 mM, it is called subclinical hypocalcemia. If the blood Ca^2+^ concentration of dairy cows is lower than 0.7 mM without the supplement, it is called clinical hypocalcemia, which can result in reduced feed intake, poor rumen and intestine motility, an increased risk of a displaced abomasum, reduced milk yield, an increased susceptibility to infectious diseases, and an increased risk of early lactation removal from the herd [10,11]. The loss of a single cow can reach thousands of dollars, mainly in feeding and milk production. Thus, it is of great importance to detect the blood Ca^2+^ content to evaluate hypocalcemia.

Currently, there is no commercial device for Ca^2+^ detection with a low cost, portability, and high precision, and is easy to use. If a veterinarian wants to know the Ca^2+^ level of a dairy cow, he has to send the samples to a professional analysis organization, which is time-consuming, high-cost, and not real-time. It is difficult to meet the requirements of low-cost and fast detection. According to the preliminary research, the Ca^2+^ content accounts for about half of the blood Ca in dairy cow [12]. The electrochemical biosensor [13] has gained the most attention in the analysis field, because its outstanding advantages include a rapid and effective response, being simple to use, low cost, and miniaturization [14], which can measure the blood Ca^2+^ with simple treatment.

A DNAzyme is a DNA molecule with catalytic activity [15], mainly consisting of two parts: an enzyme strand composed of one catalytic core and two arms, and a substrate strand with a single RNA linkage (rA). When the target substance binds to the enzyme, the substrate strand can be cleaved at the rA site [16]. In the past two decades, many DNAzymes have been developed that show excellent selectivity and sensitivity to the metal ions, such as Na^+^ [17], K^+^ [18], Ag^+^ [19], Hg^2+^ [20], Mg^2+^ [21], Cu^2+^ [22], Pb^2+^ [23], and Cr^3+^ [24]. Owing to their excellent stability, cost-effectiveness, high catalytic efficiency, and programmability [25], many DNAzyme-based biosensors have been explored for metal ion determination [26,27]. Zhou [28] previously studied a DNAzyme named EtNa that can be cleaved by Na^+^ and Ca^2+^. To improve the specificity, they further optimized the structure of EtNa to produce EtNa-C5T, which exhibited higher activity and better Ca^2+^ selectivity than the original EtNa. Considering the metal concentration difference (500-fold), the cleavage rate of EtNa was 11,200-fold higher with Ca^2+^ than with Na^+^, whereas that of EtNa-C5T was 257,000-fold higher with Ca^2+^ than with Na^+^ [29]. However, the cleavage efficiency of EtNa-C5T was easily affected by the pH that showed a positive correlation. For real samples, the pH values are different, which might affect the detecting accuracy of electrochemical biosensors.

In this work, an electrochemical biosensor was fabricated using a glassy carbon electrode, single-walled carbon nanotubes, and EtNa-C5T, but the electrochemical response was weak. To improve the sensitivity and amplify the impedance signal, the structure and length of EtNa-C5T were designed and optimized. In addition, the electrochemical biosensor was combined with a pH meter to form an electrochemical device, and the data at various pH values were used to build a mathematical model. The proposed method can simplify the pH adjustment during sample pretreatment and measure Ca^2+^ concentrations accurately.

## 2. Materials and Methods

### 2.1. Materials and Reagents

Single-walled carbon nanotubes were purchased from Jiangsu Xianfeng Nanomaterial Technology Co., Ltd. (Nanjing, China). Tween 20 (98%), 3-Aminopropyltriethoxysilane (APTES, 99%), and ethanolamine (EA, 99%) were provided by Thermo Fisher Technology Co., Ltd. (Beijing, China). NaNO_3_, KNO_3_, Mg(NO_3_)_2_, Ca(NO_3_)_2_, Cu(NO_3_)_2_, Fe(NO_3_)_2_, Zn(NO_3_)_2_, Fe(NO_3_)_3_, and Sn(NO_3_)_2_ were purchased from Sigma Aldrich Company (Beijing, China). N, N-Dimethylformamide (DMF), and ethanol were bought from Macklin Inc. (Shanghai, China). N-Hydroxysuccinimide 1-pyrene butyric acid (PBASE) was obtained from Invitrogen Company (Tianjing, China). Ethanol, hydrochloric acid (HCl), and acetic acid (CH_3_COOH) were obtained from Beijing Chemical Industry Co., Ltd. (Beijing, China). Tris(hydroxymethyl)aminomethane (Tris) was purchased from Titan Scientific Co., Ltd. (Shanghai, China). The standard stock solution of Ca^2+^ was offered by the China National Research Center for Reference Materials (Beijing, China) and diluted to different Ca^2+^ concentrations. All of the chemicals and reagents in this study were of analytical grade, which were used without further purification. Ultrapure water was produced by a Millipore Milli-Q system (18.2 MΩ cm^−3^). Both Tris-HCl buffer and potassium acetate were chosen as the buffer solution. A dry powder of oligonucleotides was synthesized by Sangon Biotech Co., Ltd. (Shanghai, China), which was dispersed in sterilized ultrapure water to 50 nM. The oligonucleotide sequences are listed in Table 1.

### 2.2. Apparatus and Measurements

A field emission scanning electron microscope (FE-SEM SU8040) was used to obtain SEM images at an accelerating voltage of 10 kV. The UV-visible absorption spectra of GCEs modified with different materials were recorded using a Beckman Kurt DU800 UV-visible spectrophotometer (Kraemer Boulevard Brea, CA, USA) at wavelengths ranging from 200 nm to 800 nm. Raman spectra were measured through Renishaw inVia with an imaging microscope (532 nm diode and Ar ion lasers). A portable pH meter (Testo 206PH) was bought from Testo AG (Beijing, China). The electrochemical measurements included cyclic voltammetry (CV) and electrochemical impedance spectroscopy (EIS), which were performed using an electrochemical cell on a CHI 760E electrochemical workstation (Shanghai Chenhua Instrument, Shanghai, China). A three-electrode system consisted of a modified glassy carbon electrode (GCE/SWNT/DNAzyme, *Φ* = 3 mm), a platinum wire, and Ag/AgCl (saturated with KCl), which served as the working electrode, counter electrode, and reference electrode, respectively. The CV measurement was swept from −0.2 V to +0.6 V with a scan rate of 50 mV s^−1^, and the frequency range of electrochemical impedance spectroscopy (EIS) ranged from 10^5^ to 0.1 Hz with the potential of 0.20 V (versus Ag/AgCl) and the amplitude of 5 mV, which were carried out in a mixture solution of 5.0 mM of [Fe(CN)_6_]^3−/4−^ and 0.1 M KCl. A centrifugal machine (SN-LSC-40) was purchased from Shanghai Shangyi Instrument Equipment Co., Ltd. (Shanghai, China).

### 2.3. Fabrication of the Biosensor

Single-walled carbon nanotubes (SWNTs) were carboxylated according to the following protocol. Briefly, the SWNTs were refluxed with a mixed solution of concentrated HNO_3_ and H_2_SO_4_ (*v*/*v* = 1:3) for 6 h. After that, the SWNTs were rinsed with enough Milli-Q purified water to reach a neutral pH. The treated SWNTs were placed in a vacuum oven and heated at 80 °C for 60 min. The SWNTs (1 mg) were dissolved in 2 mL of DMF to form a homogeneous solution.

**Figure 1:**
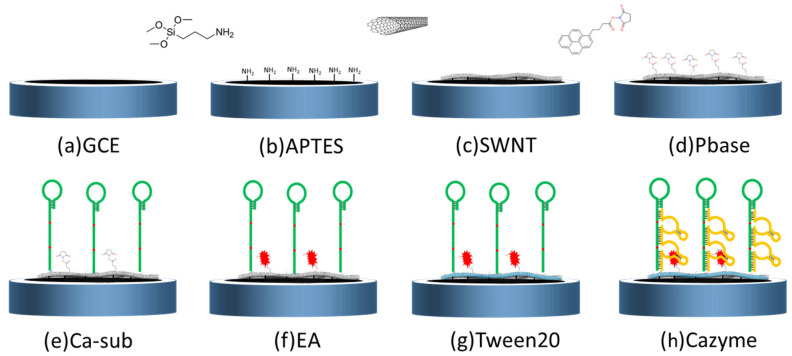
GCE functionalized with APTES, SWNT, Pbase, Ca_sub, EA, Tween20, and Cazyme.

Figure 1 illustrates the modification process as follows. First, a bare GCE was sequentially polished using 0.3 µm and 0.05 µm of Al_2_O_3_ powder, and then sonicated in DI water several times. After that, the cleaned GCE was immersed in 5% APTES in ethanol for 30 min, and then washed with sufficient ultrapure water. The GCE surface was covered with a 3 μL SWNTs solution, annealed in air at 60 °C for 30 min, and then rinsed to remove residual SWNTs. After that, the GCE/SWNT was immersed in 6 mM of PBASE in dimethylformamide for 60 min under a normal temperature. In a high-humidity environment, the PBASE-modified GCE/SWNT was covered with a 5 μL Ca substrate solution overnight at 4 °C to immobilize the Ca substrate on the SWNT surface through the amide bonds between the amine at the 5′ end and the ester groups of PBASE. The modified electrode was immersed in 0.1 mM of EA and 0.1% Tween 20 to block the excess ester groups on the PBASE and SWNT surfaces. Finally, the Ca substrate on the SWNT surface was hybridized with CAZyme to form a GCE/SWNT/DNAzyme.

### 2.4. Electrochemical Measurements

To determine the Ca^2+^ concentration, the *R_ct_* value of the electrochemical biosensor must be calculated before and after measuring the real sample. The biosensor was covered with the real sample for 9 min, and then rinsed with sufficient water to remove the residue. After that, the biosensor was immersed in 5 mM [Fe(CN)_6_]^4−/3−^ and 0.1 M KCl, and EIS was performed. The pH of the serum or milk samples was directly measured using a Testo 206PH.

The relative resistance was calculated using the following equation:

(1) $$\mathrm{Relative resistance}=\frac{\;R_{ct0}-R_{ct}}{\;R_{ct0}}\times 100\%$$

where $R_{ct0}$ is the initial resistance value, and $R_{ct}$ is the resistance after exposure to the real sample.

### 2.5. Sensing of Real Samples

Blood and milk samples were collected from dairy cows at the China–Israel demonstration dairy farm located in southeastern Beijing. The blood samples were collected from the vein of the cow’s tail using a centrifugal tube with heparin lithium, and the supernatant was obtained after centrifugation. The supernatant of centrifugation was directly used for Ca^2+^ detection. In addition, the milk samples were collected through milking equipment and treated with HNO_3_. Briefly, 15 g of a milk sample was evaporated to dryness by using an electric furnace, and then ashed in a muffle furnace at 550 °C for 5 h. After that, the sample was mixed with 5 mL of HCl (20%). The treated solution was placed into a 50 mL volumetric flask, and the volume was fixed with Tris-HCl buffer.

## 3. Results

### 3.1. Choice of Materials

EtNa-C5T (CAZyme) as a molecular recognition probe specifically hybridizes the complementary substrate (Ca_sub) to form the DNAzyme. After exposure to Ca^2+^, CAZyme can bind to Ca^2+^, and then cleave the complementary substrate (Ca_sub) at the rA site. Due to the poor conductivity of the DNAzyme, the structure change affects the impedance response of GCE/SWNTs, which can be used for Ca^2+^ detection with high selectivity. In addition, the cleavage efficiency of CAZyme is easily affected by the pH that shows a positive correlation. Therefore, a GCE/SWNTs/DNAzyme combined with a pH meter can detect Ca^2+^ concentration at variable pH environments.

### 3.2. Characterization

Morphological analysis was performed by field emission scanning electron microscopy. The GCE/SWNT before and after being functionalized with a DNAzyme is shown in Figure S1. A dense network structure was evenly distributed on the GCE surface in Figure S1A, which indicated that SWNTs were immobilized on the GCE surface. Compared to Figure S1B, there was no obvious change, indicating that it is difficult to distinguish DNAzyme modification with SEM.

**Figure 2:**
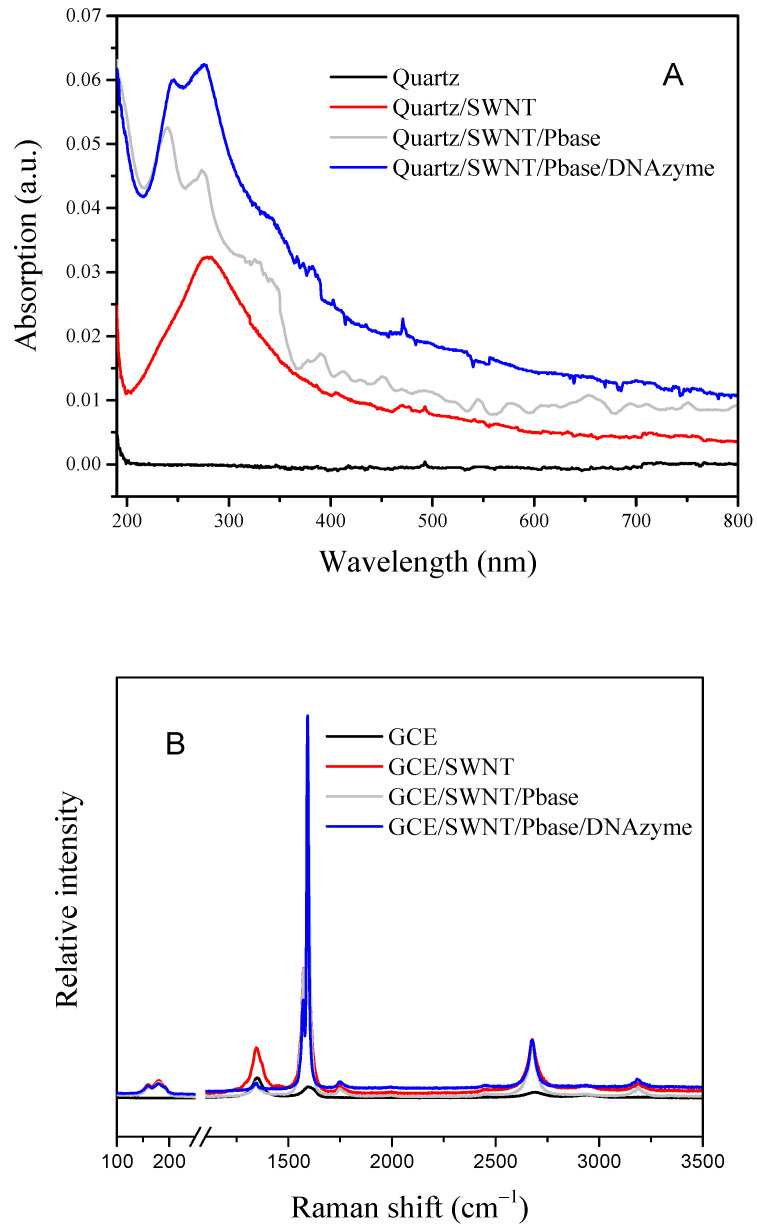
(**A**) UV-visible spectrum of blank quartz modified with SWNT, PBASE, and DNAzyme; (**B**) Raman spectrum of GCE modified with SWNT, PBASE, and DNAzyme.

The blank quartz was selected to replace the GCE because of its excellent light transmittance. Figure 2A shows the UV-visible absorption spectrum of the blank quartz functionalized with SWNTs, PBASE, and DNAzymes in the range from 190 nm to 800 nm. To reduce the interference, the absorption spectrum of blank quartz was chosen as the background, since the curve was almost a straight line. For the GCE/SWNT, a strong absorption peak was located at 280 nm, which was ascribed to SWNTs. When PBASE was modified on the GCE/SWNT, there were three absorption peaks located at 240 nm, 275 nm, and 335 nm that illustrated that PBASE had attached on the SWNT surface through CC interaction between the carbon atom of the pyrene ring and the carbon atom of SWNTs. After the DNAzyme was further functionalized on the GCE/SWNT/PBASE, there were two weak absorption peaks observed on the spectrum, contributing that the absorption peak of the DNAzyme and GCE/SWNT/PBASE was superimposed because of the UV-visible absorption peak of the DNAzyme at 260 nm. As shown in Figure 2B, Raman spectroscopy was used to investigate the change of different GCE-modified materials using 532 nm laser excitation from 100 to 3500 cm^−1^. For a bare GCE, three weak peaks were found at 1350, 1595, and 2687 cm^−1^. After the GCE was treated with SWNTs, then RBM, D, G, and 2D peaks were located at 180, 1350, 1595, and 2675 cm^−1^, which was consistent with the characteristic peaks of SWNT [30]. While the GCE/SWNT was treated with PBASE and a DNAzyme, the ratios of D to G markedly decreased, and G peak of the GCE/SWNT/DNAzyme shifted in the negative direction, indicating that PBASE and the DNAzyme were immobilized on the SWNT surface [31].

### 3.3. CV and EIS Characterization

**Figure 3:**
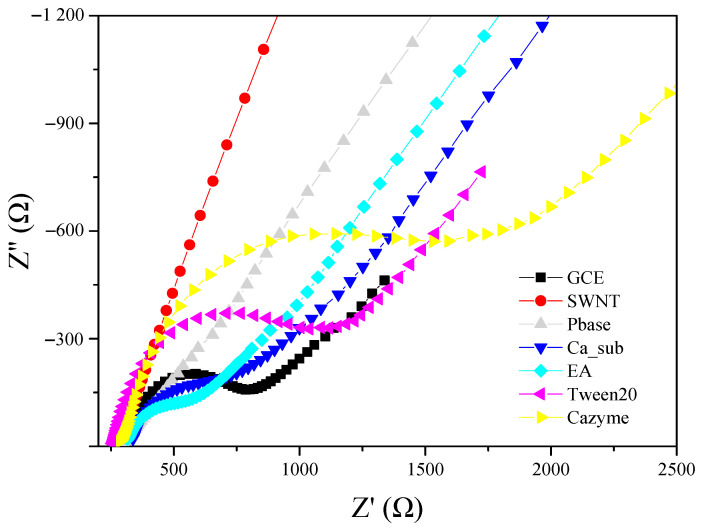
Electrochemical impedance spectra of GCE modified successively with SWNTs, PBASE, Ca_sub, EA, Tween 20, and CAZyme obtained at frequencies ranging from 1 to 10^5^ Hz (potential = 0.2 V) in 5 mmol/L [Fe(CN)_6_]^3−/4−^ and 0.1 mol/L KCl.

Electrochemical impedance spectroscopy (EIS) was employed as a highly sensitive technique to monitor the interfacial properties of each modified step, which can characterize the fabrication and assembly process of a biosensor. Each modification of the GCE/SWNT/DNAzyme was monitored by EIS analysis in 5 mM [Fe(CN)_6_]^4−/3−^ and 0.1 M KCl at ambient temperature. Figure S2 shows the Randle equivalent circuit over a range of frequencies from 100 kHz to 1 Hz, including charge transfer resistance (*R*_ct_), the capacitance (*C*_dl_), electrolyte resistance (*R*_s_), and the Warburg element (*Z*_w_). In the Nyquist plot, the *R*_ct_ directly controls the transfer kinetics of the redox-probe electron at the electrode surface, corresponding to the diameter of the semicircle. As shown in Figure 3, there was a small semicircle in the impedance spectra on the bare GCE, where the *R_ct_* was about 574 Ω. While SWNT was deposited on the GCE surface, the semicircle almost decreased into a linear plot in the impedance spectra, and the *R_ct_* was zero, ascribing to the deposition that SWNT possesses excellent electrical conductivity. After PBASE was assembled on the surface of the GCE/SWNT, there was still no obvious semicircle, but the slope of the linear plot in the impedance spectra greatly decreased. The semicircle reappeared when Ca_sub was functionalized on the SWNT surface through a peptide bond, where the *R_ct_* reached 437 Ω. The main reason is the accumulation of negatively charged probe Ca_sub that introduces much stronger steric hindrance and electrostatic repulsion to the diffusion process of redox probe [Fe(CN)_6_]^3−/4−^ toward the electrode surface. When the specific sites of PBASE were blocked through EA, the semicircle diameter increased to 504 Ω. The semicircle rose again to where the *R_ct_* was 856 Ω when Tween20 was attached to the naked SWNT surface. Finally, when CAZyme was incubated on the modified electrode from the hybridization of DNAzyme, a significantly increased semicircle could be observed, where the *R_ct_* was about 1843 Ω.

**Figure 4:**
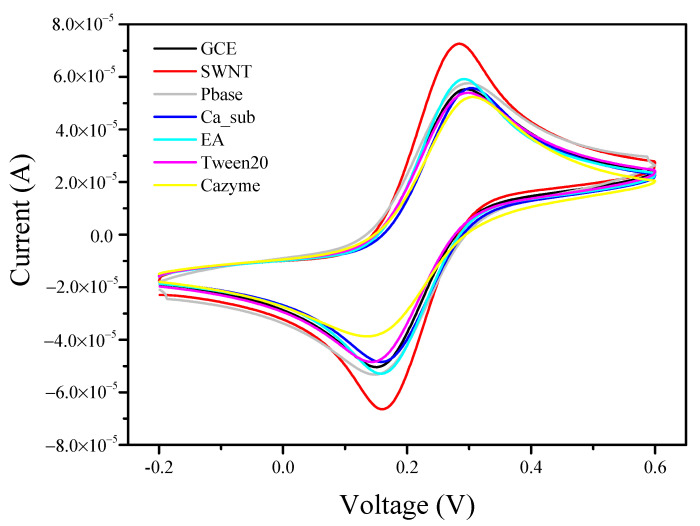
Cyclic voltammograms of the GCE before and after modification with SWNTs, PBASE, Ca_sub, EA, Tween 20, and CAZyme obtained in 5.0 mmol/L [Fe(CN)_6_]^3−/4−^ and 0.1 mol/L KCl solutions at a scan rate of 50 mV/s.

Figure 4 shows the cyclic voltammograms of the GCE functionalized successively with SWNTs, PBASE, Ca_sub, EA, Tween 20, and CAZyme in a mixed solution of 5 mmol/L [Fe(CN)_6_]^3−/4−^ and 0.1 mol/L KCl. All the CV curves exhibited a pair of well-defined redox peaks, but differences in the peak currents and the peak-to-peak separation were observed. After the SWNTs were immobilized on the GCE surface, the peak current increased significantly to 73 μA. After incubation with PBASE, the peak current decreased to 57 μA. When the negatively charged Ca_sub was assembled on the SWNT surface, the peak current was continuously reduced. After the EA and non-electroactive Tween 20 were employed to block the non-specific sites of PBASE and the bare SWNT, the peak current and peak-to-peak separation did not vary significantly. Finally, the electrode was incubated in 50 mM to form the DNAzyme, and the peak current decreased obviously. These results were consistent with the EIS results. Thus, we could preliminarily conclude that the proposed biosensor based on a DNA hydrogel was successfully prepared.

### 3.4. Optimization

To improve the sensitivity and amplify the impedance signal, several detection parameters of the GCE/SWNT/DNAzyme were optimized at different Ca^2+^ concentrations.

**Figure 5:**
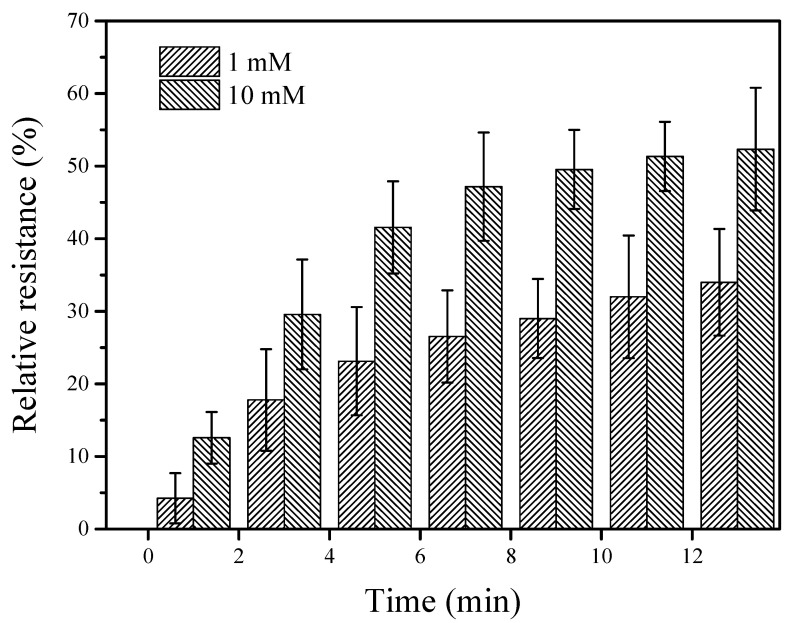
Relative resistance of the GCE/SWNT/DNAzyme after incubation in different Ca^2+^ concentrations for different times.

The incubation time can significantly affect the sensitivity of the biosensor. Figure 5 shows that the relative resistances changed with the incubation time when the GCE/SWNT/DNAzyme was immersed in different Ca^2+^ concentrations. It was clear that the relative resistance increased as the incubation time ranged from 1 to 13 min, which was attributed to the continuous cleavage of the DNAzyme by Ca^2+^. However, the growth rate of the relative resistance greatly decreased when the incubation time exceeded 7 min, especially in 10 mM of Ca^2+^. This result was mainly due to the limited DNAzyme on the GCE surface. To balance the sensitivity and detection time, 7 min was selected as the incubation time for further measurements.

**Figure 6:**
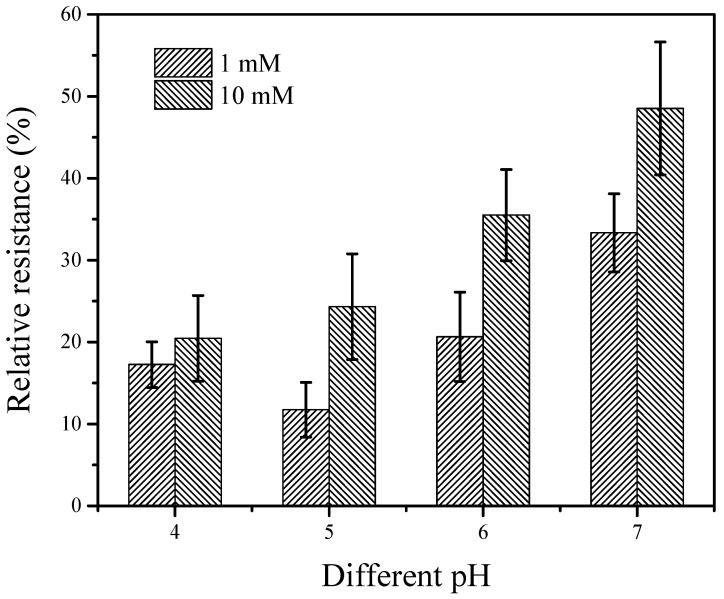
Relative resistance of the GCE/SWNT/DNAzyme after incubation in different Ca^2+^ concentrations at various pH values.

The relative resistance of the GCE/SWNT/DNAzyme was strongly affected by the pH of the electrolytic solution. Figure 6 shows the results for the GCE/SWNT/DNAzyme incubated in different Ca^2+^ concentrations at various pHs. The relative resistance increased gradually as the pH ranged from 4.0 to 7.0. One reason for this result was that the cleavage efficiency of the DNAzyme increased with the pH. In addition, hydrogen ions were absorbed on the surface of the GCE/SWNT/DNAzyme, affecting the conductivity. Therefore, it was necessary to develop a method for detecting the Ca^2+^ concentration at different pHs to improve practicality.

**Figure 7:**
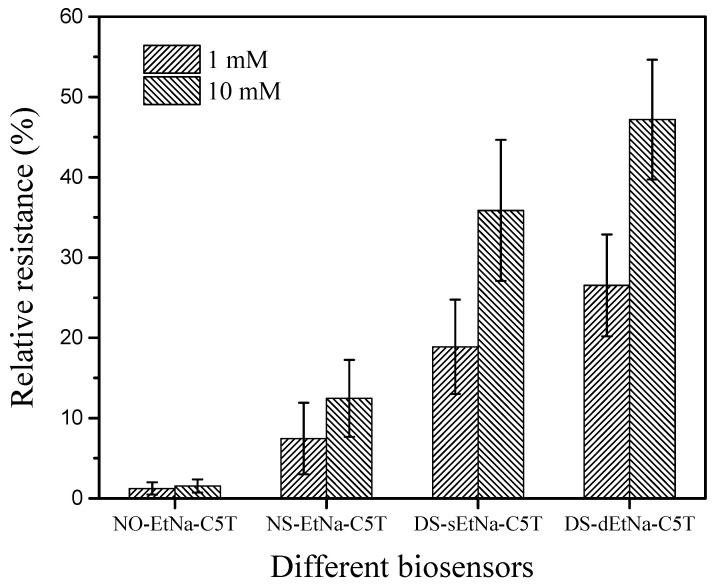
Relative resistances of GCE/SWNT/DNAzymes after incubation in different Ca^2+^ concentrations at various pH values.

The structure and length of the DNAzyme are important factors influencing the sensitivity of the GCE/SWNT/DNAzyme. As shown in Figure 7, four different DNAzymes were designed for functionalizing the GCE/SWNT, and the functionalized GCE/SWNTs were used to detect different Ca^2+^ concentrations in an electrolytic solution at pH 7.5. For the DNAzyme without rA, the relative resistance was almost zero, indicating that Ca_sub was not cleaved by the Ca/CAZyme. The relative resistances of the GCE/SWNT/DNAzyme were approximately 3.5 and 9.32 at Ca^2+^ concentrations of 1 mM and 10 mM, respectively. When the length of the DNAzyme was designed to be longer, the relative resistances reached 18 and 30 at Ca^2+^ concentrations of 1 mM and 10 mM, respectively, due to the poor conductivity of the DNA molecule. To improve the sensitivity, the two enzymes were used for the DNAzyme, and the detection response increased. Thus, the DNAzyme with two enzymes was selected as the biomaterial for this work.

### 3.5. Selectivity

**Figure 8:**
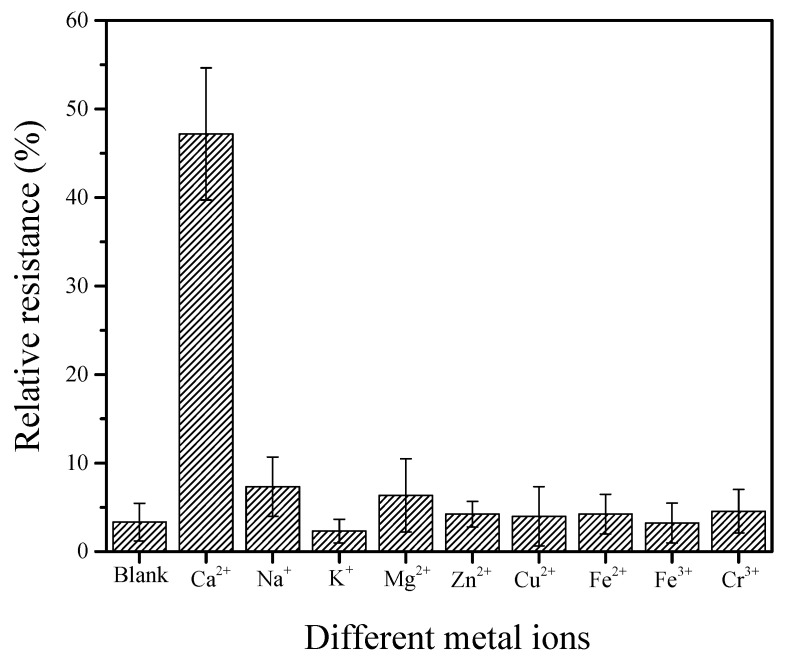
Relative resistances of the GCE/SWNT/DNAzymes before and after incubation in 10 mM solutions of different metal ions including, Na^+^, K^+^, Mg^2+^, Zn^2+^, Cu^2+^, Fe^2+^, Fe^3+,^, and Cr^3+^.

Anti-interference is an important performance factor for electrochemical biosensors. Many metal ions, such as Na^+^, K^+^, Mg^2+^, Zn^2+^, Cu^2+^, Fe^2+^, Fe^3+,^, and Cr^3+^, are present in the dairy cows’ blood and milk samples in Table S1, which might interfere with the relative resistance of a GCE/SWNT/DNAzyme. Different GCE/SWNT/DNAzymes were applied to measure 10 mM of other metal ions in an electrolytic solution at pH 7.5, and the detection results are shown in Figure 8. The relative resistance of the GCE/SWNT/DNAzymes was approximately 48 for 10 mM Ca^2+^. In comparison, the relative resistances for Na^+^ and Mg^2+^ were much higher than those for the rest of the metal ions. The four metal ion solutions had slightly higher relative resistances than the blank buffer solution, indicating that most metal ions did not interfere with the GCE/SWNT/DNAzymes.

### 3.6. Ca^2+^ Sensing

The linear relationship between the Ca^2+^ concentration and relative resistance was determined in the presence of a 10 mM Tris-HCl buffer solution via electrochemical impedance spectroscopy.

**Figure 9:**
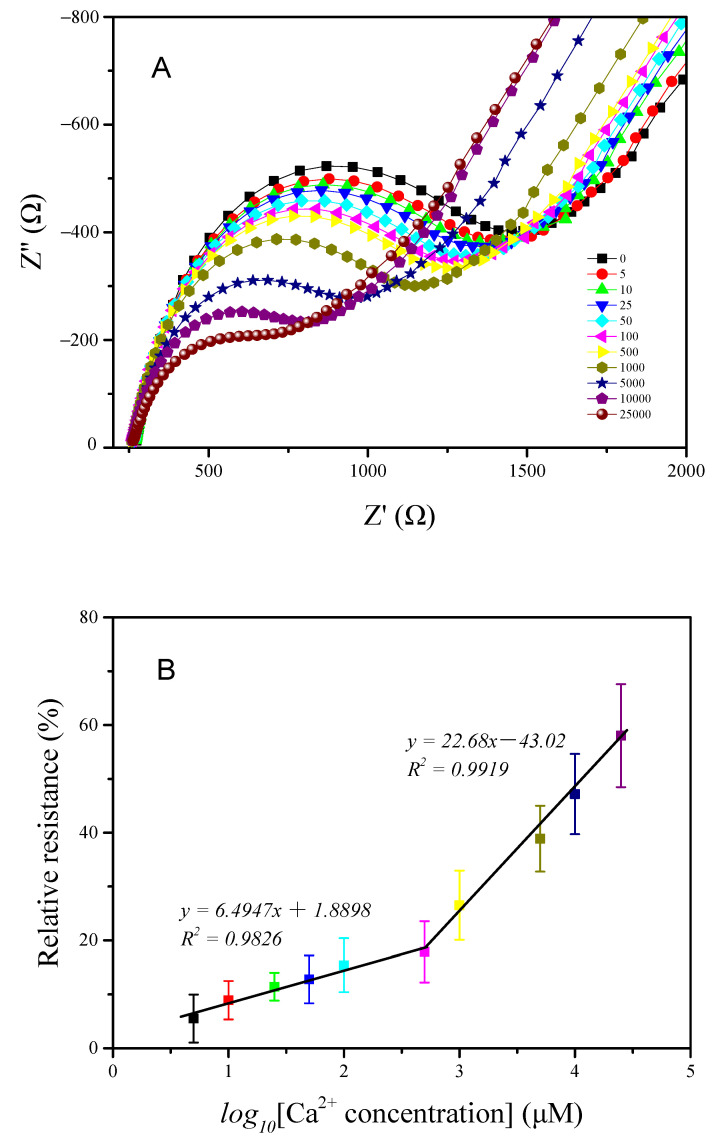
(**A**) Electrochemical impedance spectra of the GCE/SWNT/DNAzyme for different Ca^2+^ concentrations in the range of 1 μM to 25 mM; (**B**) the linear relationship between the relative resistance and the logarithm of the Ca^2+^ concentration.

The GCE/SWNT/DNAzyme measured different Ca^2+^ concentrations ranging from 0 μM to 25 mM under the optimized parameters, as shown in Figure 9A, which reveals that the semicircle diameter decreased with increasing Ca^2+^ concentrations. The calculated relative resistances are listed in Figure 9B. When the Ca^2+^ concentration was less than 500 μM, the relative resistance increased gradually, indicating that the cleavage efficiency of the DNAzyme was slow at low Ca^2+^ concentrations. The relative resistance exhibited a linear relationship with the logarithm of the Ca^2+^ concentration in the range of 5 μM to 500 μM. The linear equation was $\mathrm{Relative resistance}=1.8898+6.4947\log_{10}\left[{\mathrm{Ca}}^{2+}\right]\;\left(µM\right)$ with a linear regression coefficient of 0.991, and the detection limit was calculated to be 4.2 μM (S/N = 3). At higher Ca^2+^ concentrations, the relative resistance increased rapidly as the Ca^2+^ concentrations increased from 500 μM to 25 mM, and the linear regression equation was $\mathrm{Relative resistance}=-43.02+22.68\log_{10}\left[{\mathrm{Ca}}^{2+}\right]\;\left(µM\right)$ with a linear regression coefficient of 0.991.

### 3.7. Mathematical Model

**Figure 10:**
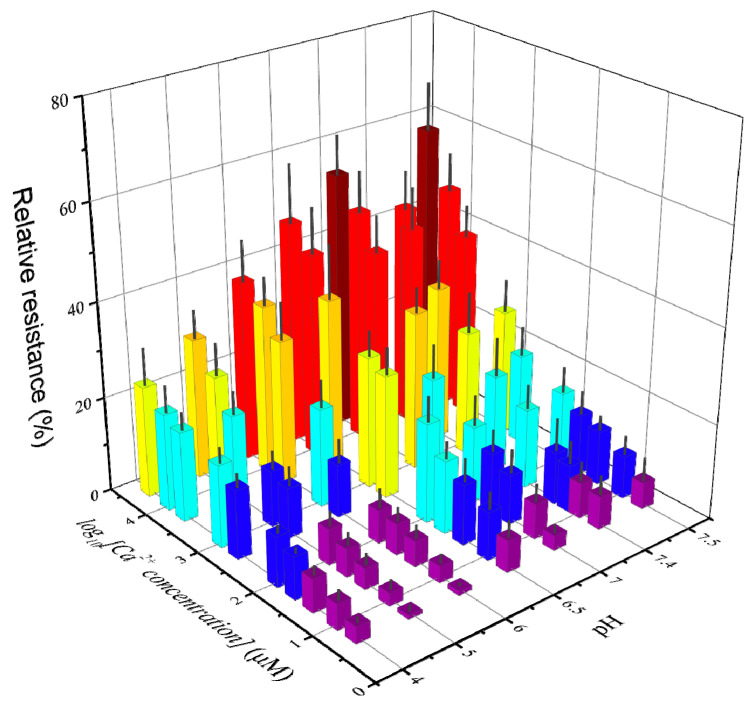
The relative resistances of the GCE/SWNTs/DNAzyme after exposure to different Ca^2+^ concentrations with various pH value. (The ratio of pH vs. Ca^2+^ concentration: 4.0–5, 4.0–10, 4.0–25, 4.0–50, 4.0–100, 4.0–500, 4.0–1000, 4.0–5000, 4.0–10000, 4.0–25000; 5.0–5, 5.0–10, 5.0–25, 5.0–50, 5.0–100, 5.0–500, 5.0–1000, 5.0–5000, 5.0–10000, 5.0–25000; 6.0–5, 6.0–10, 6.0–25, 6.0–50, 6.0–100, 6.0–500, 6.0–1000, 6.0–5000, 6.0–10000, 6.0–25000; 6.5–5, 6.5–10, 6.5–25, 6.5–50, 6.5–100, 6.5–500, 6.5–1000, 6.5–5000, 6.5–10000, 6.5–25000; 7.0–5, 7.0–10, 7.0–25, 7.0–50, 7.0–100, 7.0–500, 7.0–1000, 7.0–5000, 7.0–10000, 7.0–25000; 7.4–5, 7.4–10, 7.4–15, 7.4–25, 7.4–100, 7.4–250, 7.4–750, 7.4–2500, 7.4–7500; 7.5–5, 7.5–10, 7.5–25, 7.5–50, 7.5–100, 7.5–500, 7.5–1000, 7.5–5000, 7.5–10000, 7.5–25000.) The error bars indicate the standard deviations of five biosensors.

The pH of the electrolyte solution can affect the relative resistance of a GCE/SWNT/DNAzyme and thus interfere with the detection accuracy. To overcome this shortcoming, the GCE/SWNT/DNAzyme was coupled with a pH meter to fabricate an electrochemical sensor array that could process detection data using different mathematical models, as shown in Figure 10.

The *X* and *Y* matrices were pretreated using the following equations:

(2) $$\begin{array}{c} X=\left[1,\;x_{1},\;x_{2},\;x_{1}{}^{2},\;x_{2}{}^{2},x_{1}\times x_{2}\right]\\ Y=\log_{10}\left[C\right] \end{array}$$

where $x_{1}$ and $x_{2}$ represent the relative resistance of the GCE/SWNT/DNAzyme and the pH value, respectively, and *C* is the Ca^2+^ concentration.

#### 3.7.1. Stepwise Linear Regression

Stepwise linear regression (SLR) [32], one of several linear regression methods, is a statistical technique that selects independent variables to predict a response variable’s outcome. It can be applied to the training dataset for the Ca^2+^ concentration using statistical methods to remove the redundant variables. The basic idea is to add variables or remove them from a model step by step while performing an F-test and t-test for the selected variables individually. Variables that do not significantly change the SLR are removed to ensure that each significant variable is included. This is an iterative process that repeats until significant variables are no longer added to the SLR, and independent variables with negligible contributions are no longer removed.

First, each of the independent variables is related to the dependent variable, the value of which is calculated by the F-test.

(3) $$Y=\beta_{0}+\beta_{i}X_{i}+\in ,\;i=1,\cdots ,p$$

(4) $$F_{i_{1}}^{\left(1\right)}=max\{F_{1}^{\left(1\right)},\cdots ,F_{1}^{\left(1\right)}\}$$

where Y is the dependent variable, $X_{i}$ are the independent variable, p is the number of the independent variables, $\beta_{i}$ are regression coefficients, and $F_{i_{1}}^{\left(1\right)}$ is the value of F-test or T-test. If $F_{i_{1}}^{\left(1\right)}$ was greater than a certain significant level, $X_{i_{1}}$ was selected for the SLR.

Then, different sets of independent variables were chosen, such as $\{X_{i_{1}},X_{1}\},\cdots ,\{X_{i_{1}},X_{i_{1}-1}\},\{X_{i_{1}},X_{i_{1}+1}\},\cdots ,\{X_{i_{1}},X_{p}\}$, and a binary regression with the dependent variable was performed.

(5) $$F_{i_{2}}^{\left(2\right)}=max\left\{F_{1}^{\left(2\right)},\cdots ,F_{i_{1}}^{\left(2\right)},F_{i_{1}+1}^{\left(2\right)},\cdots ,F_{p}^{\left(2\right)}\right\}$$

where $F_{i_{2}}^{\left(2\right)}$ is the value of the F-test or T-test. If $F_{i_{2}}^{\left(2\right)}$ was greater than a certain significance level, $X_{i_{2}}$ was selected for the SLR. The rest of the independent variables were determined in turn. 

**Figure 11:**
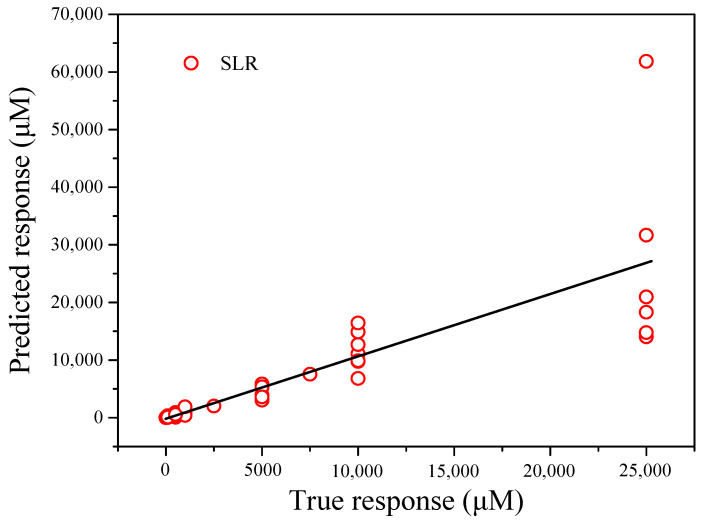
Predicted Ca^2+^ concentrations obtained by stepwise linear regression vs. the actual Ca^2+^ concentrations.

The *X* matrix was chosen as the independent variable, and the *Y* matrix was selected as the dependent variable. The matrices were processed using SLR. The actual response vs. the predicted response is shown in Figure S3 and Figure 11. Based on the computation of the model of statistics, the coefficients of determination (R-squared) for the actual and predicted Ca^2+^ concentrations was approximately 0.95. The root means square error (RMSE) of the true and predicted Ca^2+^ concentrations was 0.26127. The mean square error (MSE) and mean absolute error (MAE) were 0.068261 and 0.19161, respectively.

#### 3.7.2. Neural Network Fitting

Neural network fitting (NNF) [33] is a non-parametric non-linear method that combines the advantages of neural networks and regression. The most important feature is that the mapping equation between the input and output does not need to be determined during learning. The NNF procedure can be divided into three layers, namely input, hidden, and output layers.

**Figure 12:**
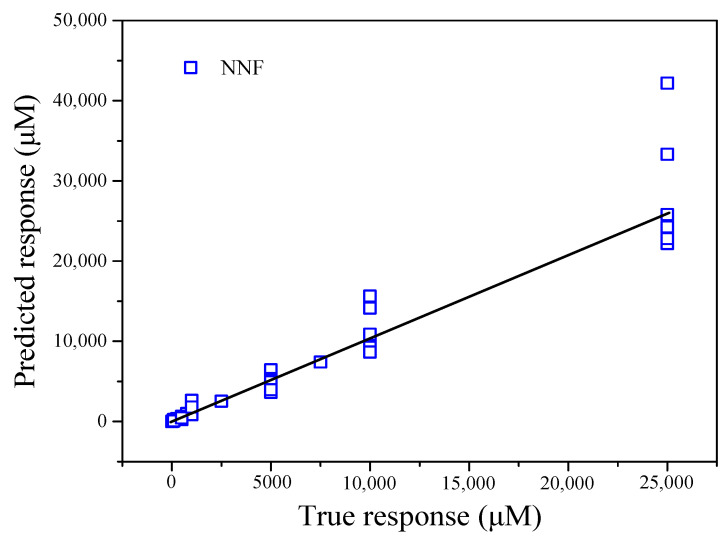
Predicted Ca^2+^ concentrations obtained by the established NNF model vs. the actual Ca^2+^ concentrations.

The *X* matrix was chosen as the input layer, the corresponding *Y* matrix was selected as the output layer, and the mapping relationship between the input and output layers was established through the hidden layer. The whole dataset included 70 samples, which were divided randomly into three groups: the proportions of the training, validation, and test sets were 70%, 15%, and 15%, respectively. As shown in Figure S4, the number of hidden layer nodes was set to 10, based on the optimization of the hidden nodes with the Levenberg–Marquardt (LM) algorithm. The predicted Ca^2+^ concentrations versus the actual Ca^2+^ concentrations are shown in Figure S5 and Figure 12. An RMSE of 0.386, MSE of 0.1481, and R-squared value of 0.9926 were obtained for the training dataset.

#### 3.7.3. Gaussian Process Regression

Gaussian process regression [34,35] is a non-parametric statistical method for functional regression analysis that focuses on problems involving functional response variables, as well as mixed functional and scalar covariates. GPR has several advantages, for example, it works well with small datasets and can provide uncertainty measurements for predictions.

With the dataset $D=\left\{X,\;Y\right\}={\left\{x_{i},y_{i}\right\}}_{i=1}^{n}$, the regression model can be formulated as

(6)  y=f(x)+ε

where f(·) denotes an unknown regression function, and ε is the Gaussian noise with a zero mean and variance $\sigma_{n}^{2}$. From the funtion space view, the Gaussian process is completely specified by its mean function m(x) and covariance function $C\left(x,x^{\prime }\right)$ from the function space view, which are defined as follows:

(7)  m(x)=E[f(x)]

(8) $$C\left(x,x^{\prime }\right)=E[\left(f\left(x\right)-m\left(x\right)\right)(f\left(x^{\prime }\right)-m\left(x^{\prime }\right)]$$

Then, the Gaussian process can be described by:

(9) $$f\left(x\right)\sim GP\left(m\left(x\right),\;C\left(x,\;x^{\prime }\right)\right)$$

Usually, the data are normalized for notation simplicity, and then the output observations follow a Gaussian distribution as

(10) $$y\sim GP\left(0,\;C\left(x,\;x^{\prime }\right)\right)$$

When a query input x_*_ is received, the joint distribution of the training outputs y and test output y^*^ based on the previous test is

(11) $$\left[\begin{array}{c} y\\ y_{x} \end{array}\right]\sim N\left(0,\left[\begin{array}{cc} c & k^{*}\\ k^{T} & C\left(x_{*},x_{*}\right) \end{array}\right]\right)$$

where $k_{*}={\left[C\left(x_{*},x_{1}\right),\;\cdots ,C\left(x_{*},x_{n}\right)\right]}^{T}$. Then, the prediction output ($y_{*}$) and variance ($\sigma_{*}{}^{2}$) can be given as:

(12) $$\;\{\begin{array}{c} y^{*}=k_{*}^{T}\\ \sigma_{*}{}^{2}=C\left(x_{*},x_{*}\right)-k_{*}^{T}C^{-1}k_{*} \end{array}$$

**Figure 13:**
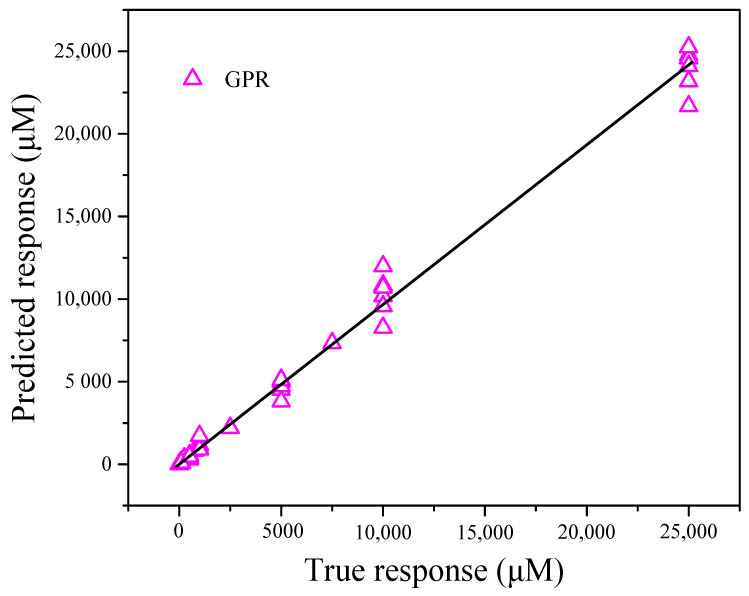
Predicted Ca^2+^ concentrations obtained by the established GPR model vs. the actual Ca^2+^ concentrations.

The *X* matrix was chosen as the workspace variable, and the *Y* matrix was selected as the response. Cross-validation was set as 10 folds. The predicted concentrations obtained after modelling the training dataset vs. the actual concentrations of Ca^2+^ are shown in Figure S6 and Figure 13. The RMSE, MSE, MAE, and R-squared values were calculated as 0.23753, 0.056421, 0.18097, and 0.96, respectively.

### 3.8. Comparison of Detection Performance

The parameters of different sensors for Ca^2+^ determination are listed in Table 2. Clearly, sensors based on different principles have various advantages during usage. Most of the ion-selective electrodes based on membrane potentials had a fast detection time and wide linear range, but the selectivity was lower than the GCE/SWNTs/CAZyme. For spectroscopic and electrochemical methods, the detection time and pH range had no obvious advantages, but the linear range and detection limit were similar to those of the ion-selective electrode. For this work, the electrochemical device (GCE/SWNTs/CAZyme and pH meter) overcome the shortcoming that biosensors can only detect Ca^2+^ in a specific pH environment, making it suitable for different biological samples without the need for complicated pretreatment.

### 3.9. Detection of Ca^2+^ in Real Sample

A comparison of the three methods revealed significant differences in the model parameters. The RMSE and MSE values for GPR were lower than those for SLR and NNR. Based on the formula calculations, the R-squared value for NNR was much higher than those for SLR and GPR. In addition to the inverse transformation of the Ca^2+^ concentrations for each method, these results are shown in Figure 11, Figure 12, and Figure 13. Therefore, GPR was chosen as the optimal analysis model.

The electrochemical device was used to investigate three different blood and milk samples collected from dairy cows. As previously described, the blood and milk samples were pretreated following the protocol in Section 2.5. The extracted samples were studied by the proposed method and atomic absorption spectrometry (AAS), and the detection results are shown in Table 3. We found that the detection accuracy of the proposed method was lower than that of the AAS. The average recoveries ranged from 93.42% to 113.28%, showing that the electrochemical device can be used to determine Ca^2+^ concentrations in the blood and milk samples. A t-test was used to analyze the results of the two methods, and *p* = 0.956 > 0.05. In addition, the proposed method only requires a portable instrument that is inexpensive and easy to use. These results confirmed that the proposed method could be successfully used to determine Ca^2+^ concentrations in blood and milk samples of dairy cows.

## 4. Conclusions

In this study, a novel method was proposed to establish an electrochemical device using an electrochemical biosensor and a pH meter, which overcome the electrochemical biosensor interfering with the pH in an analyte. The electrochemical biosensor was fabricated by modifying a glassy carbon electrode with SWNTs and a Ca^2+^-cleaved DNAzyme. The sensitivity of the GCE/SWNT/DNAzyme was improved by changing the structure and length of the DNAzyme. The linear range was from 5 μM to 25 mM, with a detection limit of 4.2 μM. The GCE/SWNT/DNAzyme was combined with a pH meter to form an electrochemical device, and the data of different Ca^2+^ concentrations at different pHs were processed using three different mathematical models, namely MLR, NNF, and GPR. Among them, GPR showed a comparatively excellent performance in predicting the Ca^2+^ concentration at pH values ranging from 4.0 to 7.5. The prepared electrochemical device was applied to detect Ca^2+^ in blood and milk samples with good results.

## Figures and Tables

**Table 1:** Sequences of the modified DNA.

DNA Name		Sequences and Modifications (Starting from 5 Terminal ′)
Ca_sub	NO-substrate	NH_2_-(CH_2_6)GCGGTAGAAGGATATCACTGAGCACTG
	NS-substrate	NH_2_-(C6)GCGGTAGAAGG/rA/TATCACTGAGCACTG
	DS-substrate	NH_2_-(C6)GCGGTAGAAGG/rA/TATCACTGAGCACTGGG/rA/TAAGCGG TAGAACTCACAATGTATAATGCGCGCATTATACATTGTGAGT
CAZyme	EtNa-C5T	CAGTGCTCAGTGATTGTTGGAATGGCTCATGCCACACTCTTTTCTACCGC
	sEtNa-C5T	TCTACCGCTTATCCCAGTGCTCAGTGATTGTTGGAATGGCTC ATGCCACACTCTTTTCTACCGC
	dEtNa-C5T	TCTACCGCTTTGTTGGAATGGCTCATGCCACACTCTTCAGTGCT CAGTGATTGTTGGAATGGCTCATGCCACACTCTTTTCTACCGC

**Table 2:** Comparison of the performances of electrochemical device and other reported sensors for Ca^2+^ determination.

Sensor	Method	pH	Time (min)	Linear Range (μM)	LOD (μM)	Ref.
CD-EGTA	Fluorescent	7	240	15 ∼ 300 μM	0.38 μM	[8]
DNAzyme/SWNT/FET	Current	6.9	9	10 ∼ 1000	7.2	[27]
DLLME	UV-visible	12.8	30	1.5 ∼ 37.5	0.425	[36]
SD-ISO	Fluorescence	6.0–8.0	5	10 ∼ 10^6^	9.3	[37]
Ca-ISE	Potential	-	1.67	10^−2.5^ ∼ 10^−1.5^ M	-	[38]
Ca^2+^-SCISE	Potential	-	1	10^−1^ ∼ 10^4^	5	[39]
Fe_2_O_3_-ZnO NRs/FET	Current	7.6	-	0.01 ∼ 3.0 mM	0.05	[40]
Electrochemical device	EIS	4.0–7.5	7	5 ∼ 2.5 × 10^4^	4.2	This work

CD-EGTA: carbon dot-ethylenebis(oxyethylenenitrilo)tetraacetic acid; ISO: ion-selective optode; SD: solvatochromic dye; ISE: potentiometry based on a calcium-selective electrode; SCISE: solid-contact calcium-selective electrode.

**Table 3:** Ca^2+^ concentrations in the real samples determined by the electrochemical device and AAS.

Sample		Electrochemical Device (mM)	AAS (mM)	Recovery (%)
Blood	1	0.78	0.73	106.85
	2	1.15	1.12	102.68
	3	0.81	0.88	92.05
	4	1.09	1.17	93.16
Milk	1	27.32	29.20	93.56
	2	31.34	28.45	105.34
	3	21.87	22.78	96.01
	4	34.34	37.87	90.68

## Data Availability

Not applicable.

## Supplementary Materials

The following are available online at https://www.mdpi.com/article/10.3390/nano12010004/s1, Figure S1: SEM image of GCE/SWNT/DNAzyme, Figure S2: Randle equivalent circuit, Figure S3: predicted Ca^2+^ concentration against true Ca^2+^ concentration using the stepwise linear regression, Figure S4: overall structure of the NNF model with the LM algorithm, Figure S5: predicted Ca^2+^ concentration against true Ca^2+^ concentration using the established NNF model, Figure S6: predicted Ca^2+^ concentration against true Ca^2+^ concentration using the established GPR model, Table S1: Related indexes in dairy blood.
