# Supplementary material for: DNAzyme-Amplified Electrochemical Biosensor Coupled with pH Meter for Ca2+ Determination at Variable pH Environments
Source: Nanomaterials (Basel). 2021 Dec 21;12(1):4. doi: 10.3390/nano12010004 (PMC8746961; doi:10.3390/nano12010004)
Supplement: Supplementary file 1 [file nanomaterials-12-00004-s001.zip › nanomaterials-1476812-supplementary.pdf]

Supporting Information

DNAzyme-Amplified Electrochemical Biosensor Coupled with pH Meter for Ca^{2+} Determination at Variable pH Environments

Hui Wang [1], Fan Zhang [1,2], Yue Wang [1,3], Fangquan Shi [4], Qingyao Luo [1], Shanshan Zheng [1], Junhong Chen [5], Dingzhen Dai [5], Liang Yang [1,*], Xiangfang Tang [1,*] and Benhai Xiong [1,*]

[1] State Key Laboratory of Animal Nutrition, Institute of Animal Science, Chinese Academy of Agricultural Sciences, Beijing 100193, China; wanghui_lunwen@163.com (H.W.); zhangfan19@139.com (F.Z.); wangyue9313@163.com (Y.W.); luoqingyao@caas.cn (Q.L.); zhengshanshan@caas.cn (S.Z.)
[2] College of Animal Science and Technology, China Agricultural University, Beijing 100193, China
[3] College of Animal Science and Technology, Northwest A&F University, Yangling 712100, China
[4] Animal Husbandry and Veterinary Station of Xihe County, Longnan City, Gansu 742100, China; sfquan@163.com
[5] Department of Animal Science and Technology, Jinling Institute of Technology, Nanjing 211169, China; chenjunhong@jit.edu.cn (J.C.); dzdai@163.com (D.D.)
* Correspondence: yangliang@caas.cn (L.Y.); tangxiangfang@caas.cn (X.T.); xiongbenhai@caas.cn (B.X.). Tel.: +86-010-62811680 (B.X.)

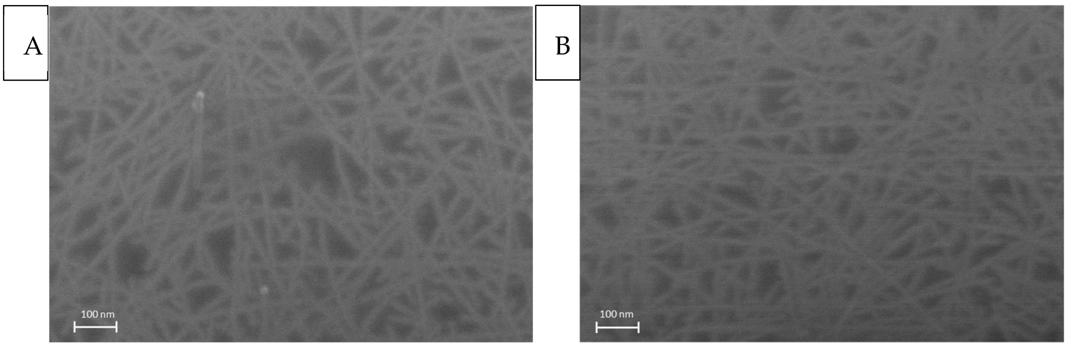

Figure S1. SEM images of GCE/SWNT(A) and GCE/SWNT/DNAzym(B)

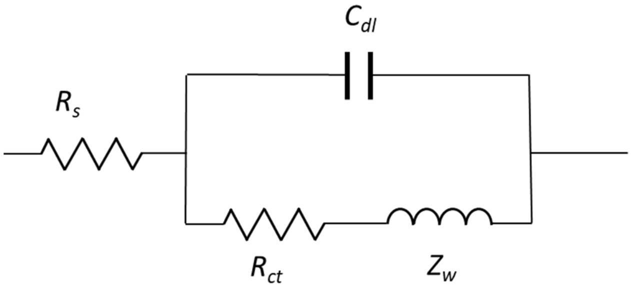

Figure S2. Randles equivalent circuit

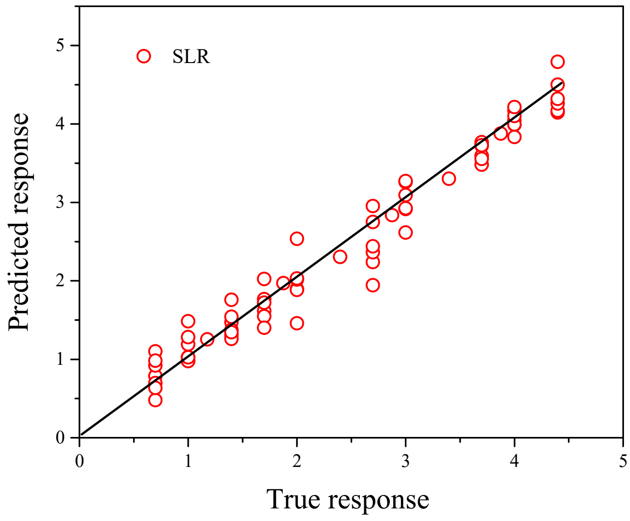

Figure S3. Predicted Ca^{2+} concentration against true Ca^{2+} concentration using the stepwise linear regression.

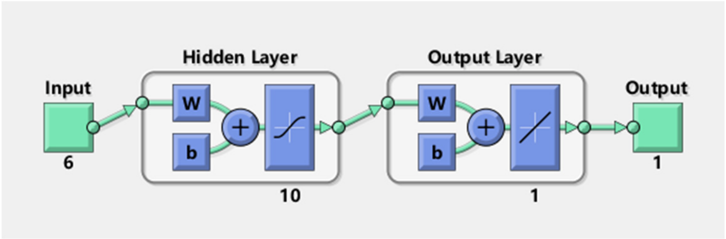

Figure S4. Overall structure of the NNF model with the LM algorithm.

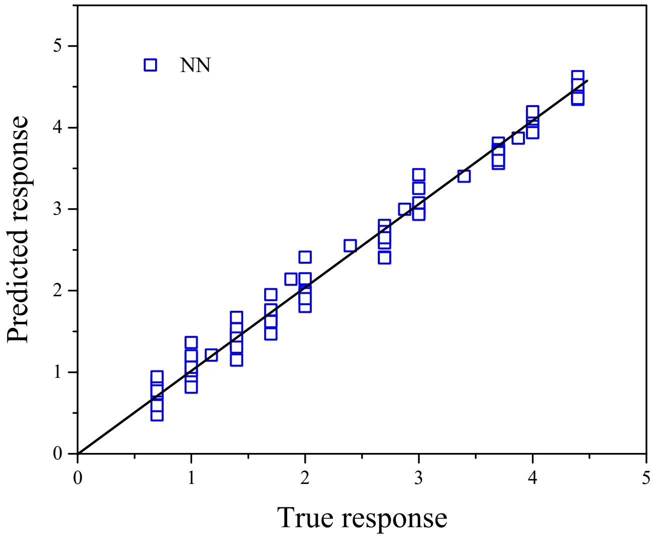

Figure S5. Predicted Ca^{2+} concentration against true Ca^{2+} concentration using the established NNF model.

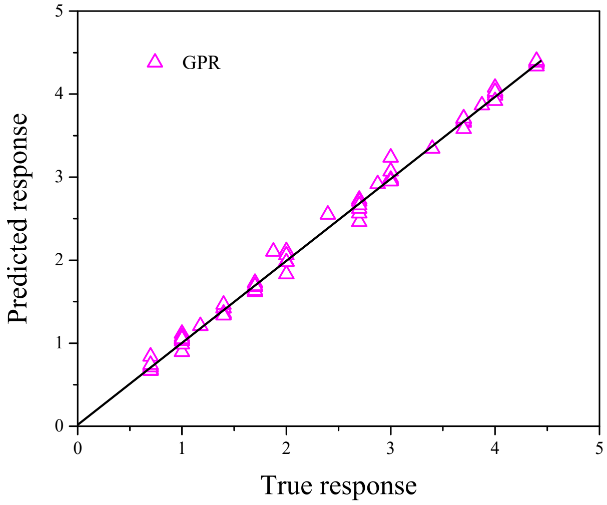

Figure S6. Predicted Ca^{2+} concentration against true Ca^{2+} concentration using the established GPR model

Table S1. Related indexes in dairy blood

Metal	Ca	Na	K	Mg	Zn	Cu	Fe	Fe	Cr
Total Value (mM)	2.1-2.8	135-148	4.0-5.8	0.7-1.2	0.012-0.018	2.3-3.1	12.8-42.9	12.8-42.9	-
